# Differential gene expression in the salivary gland during development and onset of xerostomia in Sjögren's syndrome-like disease of the C57BL/6.NOD-*Aec1Aec2 *mouse

**DOI:** 10.1186/ar2676

**Published:** 2009-04-20

**Authors:** Cuong Q Nguyen, Ashok Sharma, Byung Ha Lee, Jin-Xiong She, Richard A McIndoe, Ammon B Peck

**Affiliations:** 1 Department of Oral Biology, College of Dentistry, 1600 SW Archer Rd., University of Florida, Gainesville, FL 32610, USA; 2 Center for Biotechnology & Genomic Medicine, CBGM 1120 15th Street CA4126, Medical College of Georgia, Augusta, GA 30912, USA; 3 Department of Pathology, Immunology & Laboratory Medicine, College of Medicine, 1600 SW Archer Rd., University of Florida, Gainesville, FL 32610, USA; 4 Center for Orphan Autoimmune Diseases, College of Dentistry, 1600 SW Archer Rd., University of Florida, Gainesville, FL 32610, USA

## Abstract

**Introduction:**

Recently, we reported the development of the C57BL/6.NOD-*Aec1Aec2 *mouse that carries two genetic intervals derived from the non-obese diabetic (NOD) mouse capable of conferring Sjögren's syndrome (SjS)-like disease in SjS-non-susceptible C57BL/6 mice. In an attempt to define the molecular bases underlying the onset of stomatitis sicca (xerostomia) in this C57BL/6.NOD-*Aec1Aec2 *mouse model, we have carried out a study using genomic microarray technology.

**Methods:**

By means of oligonucleotide microarrays, gene expression profiles of salivary glands at 4, 8, 12, 16, and 20 weeks of age were generated for C57BL/6.NOD-*Aec1Aec2 *male mice. Using Linear Models for Microarray Analysis and B-statistics software, 480 genes were identified as being differentially expressed (*P *< 0.01 and Q < 0.0001) during the development of SjS-like disease in the salivary glands.

**Results:**

The 480 genes could be arranged into four clusters, with each cluster defining a unique pattern of temporal expression, while the individual genes within each cluster could be grouped according to related biological functions. By means of pair-wise analysis, temporal changes in transcript expressions provided profiles indicating that many additional genes are differentially expressed at specific time points during the development of disease. Multiple genes reportedly showing an association with autoimmunity and/or SjS, in either humans or mouse models, were found to exhibit differential expressions, both quantitatively and temporally. Selecting various families of genes associated with specific functions (for example, antibody production, complement, and chemokines), we noted that only a limited number of family members showed differential expressions and these correlated with specific phases of disease.

**Conclusions:**

Taking advantage of known functions of these genes, investigators can construct interactive gene pathways, leading to modeling of possible underlying events inducing salivary gland dysfunction. Thus, these different approaches to analyzing microarray data permit the identification of multiple sets of genes of interest whose expressions and expression profiles may correlate with molecular mechanisms, signaling pathways, and/or immunological processes involved in the development and onset of SjS.

## Introduction

The salivary gland system, comprised of the parotid, submandibular, sublingual, and minor salivary glands, secretes fluids rich in proteins that are critical for the maintenance of oral health. Saliva functions to buffer the acidification produced by bacteria residing within the oral cavity, replace ions, moisten food, and lubricate the oral cavity and esophagus (important for taste, speech, and swallowing). Saliva also contains digestive enzymes like amylase, anti-microbial substances like secretory immunoglobulins, histatins, and splunc, and growth factors like epidermal growth factor (EGF). While there are multiple underlying causes for decreased secretions of saliva, one of the more severe causes of xerostomia sicca, or dry mouth disease, results from an autoimmune disease, referred to as Sjögren's syndrome (SjS), in which the immune system targets initially the salivary and/or lacrimal glands [1-3].

Despite expanding efforts to define the genetic, environmental, and immunological bases of SjS, the underlying etiology of this disease remains ill defined. Over the past 20 years, a variety of mouse strains have been developed to study the immuno-pathophysiological nature of SjS. Based on results of studies using non-obese diabetic (NOD) mice and various single-gene knockout congenic partner strains of NOD, we have postulated that the development and onset of autoimmune exocrinopathy can be divided into at least three distinct temporal, yet consecutive and overlapping, phases. In phase 1, aberrant genetic, physiological, and biochemical activities, resulting presumably from retarded salivary gland development and increased acinar cell apoptosis, occur between 6 and 10 weeks of age. In phase 2, occurring around 10 to 18 weeks of age, exocrine gland injury is observed in conjunction with the appearance of leukocytic infiltrates and formation of lymphocytic foci consisting mostly of T- and B-cell aggregates. In phase 3, an overt clinical disease occurs and is defined by measurable loss of salivary and lacrimal gland secretory function, usually detected after 18 to 20 weeks of age [4,5]. Salivary and lacrimal gland dysfunction in SjS is currently thought to result from a combination of (a) pro-inflammatory cytokine production capable of inducing cellular apoptosis and auto-antibodies reactive with the muscarinic acetylcholine and adrenergic receptors and (b) the action of infiltrating T cells (possibly CD4^+ ^T_H_17 cells) [6], leading to a progressive loss of acinar cell mass.

Pathological changes observed in this SjS mouse model appear to occur as a consequence of altered glandular homeostasis [7]. Aberrant proteolytic activity, elevated apoptosis, downregulated EGF gene expression, and reduced α-amylase activity are commonly observed around 8 weeks of age prior to disease onset and independent of detectable autoimmunity. While the factors driving these physiological changes remain unknown, this altered glandular homeostasis is hypothesized to be the basis for why autoreactive T cells eventually attack exocrine gland tissue [8]. Thus, as anticipated during the development and onset of SjS, multiple genes, signaling pathways, molecular networks, and immunological processes will exhibit temporal expressions that may reflect their pathogenic functions. This concept has been strongly supported by our recent microarray studies of differentially expressed genes in the lacrimal glands during the development and onset of xerophthalmia in the NOD-derived C57BL/6.NOD-*Aec1Aec2 *mouse model of primary SjS [9].

Taking advantage of microarray technology to screen for temporal changes in the expression of large numbers of genes, we recently identified a set of differentially expressed genes in the salivary glands of C57BL/6.NOD-*Aec1Aec2 *mice at 8 versus 12 weeks of age, two time points covering the initial onset of detectable autoimmunity in this mouse model [10]. Results of that study identified a set of sequential activations involving several biological processes and signaling pathways conceptually important in SjS disease. During the pre-autoimmune phase, genes upregulated at 8 weeks of age encode factors associated with interferon, Toll-like receptor, and apoptotic signaling pathways highly indicative of pro-inflammatory stimuli, especially interleukin (IL)-1 and IL-18. By 12 weeks of age, the upregulated clustered genes had switched to encode factors associated with adaptive immunity, especially B-cell activation and differentiation. In the present study, we expanded this comparison of differentially expressed genes to cover the full spectrum for development and onset of SjS-like disease. Our goal has been to address the hypothesis that identification of genes exhibiting changes in expression that correlate with disease progression will provide an in-depth snapshot of molecular signaling pathways associated with noted pathophysiological alterations in the salivary glands and the subsequent onset of autoimmunity leading to salivary gland dysfunction.

## Materials and methods

### Animals

C57BL/6.NOD-*Aec1Aec2 *and C57BL/6J mice were bred and maintained under specific pathogen-free conditions within the mouse facility of the Department of Pathology with oversight by Animal Care Services at the University of Florida, Gainesville. The animals were maintained on a 12-hour light-dark schedule and provided food and acidified water *ad libitum*. Although SjS in humans is most common in post-menopausal women, male mice were used exclusively in the present study as we have not noticed differences in the salivary gland disease in male and female C57BL/6.NOD-*Aec1Aec2 *mice. Mice were euthanized at 4, 8, 12, 16, or 20 weeks of age by cervical dislocation after deep anesthetization with isoflurane. There are no indications that this procedure affects physiological function of the exocrine glands. Both the breeding and use of these animals for the present studies were approved by the University of Florida Institutional Animal Care and Use Committee.

### Preparation of RNA for detection of differentially expressed genes in microarray analyses

Salivary glands were freshly excised from individual male mice (n = 5 per age group) at 4, 8, 12, 16, or 20 weeks of age, snap-frozen in liquid nitrogen, and stored at -80°C until all glandular samples were obtained. With one lobe of each salivary gland, comprised of a submandibular, sublingual, and parotid gland minus any salivary lymph nodes, all 25 samples of total RNA from the five age groups of C57BL/6.NOD-*Aec1Aec2 *mice were isolated concurrently using the RNeasy Mini-Kit (Qiagen, Valencia, CA, USA) in accordance with the protocol of the manufacturer. To account for any asynchrony of SjS-like disease within C57BL/6.NOD-*Aec1Aec2 *male mice, the five mice in each age group were derived from at least two litters. Hybridizations were carried out with each of the 25 individual RNA samples using Affymetrix GeneChip Mouse Genome 430 2.0 Arrays in accordance with the instructions of the manufacturer (Affymetrix, Santa Clara, CA, USA). Each GeneChip contains 45,000 probe sets that analyze the expression level of over 39,000 transcripts and variants from over 34,000 well-characterized mouse genes. Microarray data have been deposited with Gene Expression Omnibus accession number [GEO:GSE15640].

### Differential gene expression analysis

Microarray data were normalized using the 'guanine-cytosine robust multi-array average' (GCRMA) algorithm and analyzed using the LIMMA (Linear Models for Microarray Analysis) package from the R Development Core Team (The R Project for Statistical Computing [11]) to perform differential expression analyses. LIMMA takes into account the correlation between replicates and uses the empirical Bayes approach, which gives stable inference for a relatively small number of arrays [12]. In this study, the 'fdr' method to adjust the *P *values for multiple testing was used to control the false discovery rate [13]. Since the data represent five equally spaced time points, multiple models were used to identify the temporal patterns of gene expression. These included the linear fit (degree = 1), quadratic fit (degree = 2), cubic fit (degree = 3), and quartic fit (degree = 4) regression models. B-statistics (the log of the odds of a gene showing either positive or negative trends over time) were calculated for each gene. Genes exhibiting a B-statistic of greater than 1.5 were considered differentially expressed in the present analysis, and this represents a greater than 82% level of probability that a gene is differentially expressed. Duplicate genes, when present, were removed and their expression levels were averaged across the duplicates.

### Verification of selected gene expression by semi-quantitative reverse transcriptase-polymerase chain reaction analysis

Aliquots of salivary gland RNA were prepared for each of the experimental time points (4, 8, 12, 16, and 20 weeks) by pooling the five individual RNA samples prepared for each age group, as described above. Each pooled aliquot then was used to synthesize cDNA. Synthesis of cDNA was carried out with 1 μg of RNA using Superscript II reverse transcriptase (Invitrogen Life Technologies, Fredrick, MD, USA) in accordance with the protocol of the manufacturer. The cDNA was quantified by spectrophotometry, and semi-quantitative polymerase chain reactions (PCRs) were performed using 1 μg of cDNA as template. After an initial denaturation at 94°C for 4 minutes, each PCR was carried out for 40 cycles consisting of 94°C for 1 minute and annealing temperatures at 60°C for 45 seconds and 72°C for 1 minute. The forward and reverse sequences of each primer set were *Akt*1, forward: AGGATGTTTCTACTGTGGGCAGCA, reverse: TGTCTCTGAACAGCATGGGACACA;* ApoE*, forward: AGATGGAGGAACAGACCCAGCAAA, reverse: TGTTGTTGCAGGACAGGAGAAGGA; *Ctsb*, forward: AGATTTGGGCGATGGCCTTCAAAC, reverse: ATGTGCTTGCTACCTTCCTCTGGT; *Fdft1*, forward: AGTCGCAAGGATGGAGTTCGTCAA, reverse: AACGTAGTGGCAGTACTTGTCCCA; and *G3pdh*, forward: GCCATCACTGCCACCCAGAAG, reverse: GTCCACCACCCTGTTGCTGCA. PCR products were size-separated by electrophoresis using 1.2% agarose gels and visualized with ethidium bromide staining. PCR band intensities were compared to *G3pdh *using the Quantity One 1-D Analysis Software (Bio-Rad Laboratories, Inc., Hercules, CA, USA). Relative band intensities were determined by dividing the intensity of the mRNA of selected genes by the density of the *G3pdh *band.

### Cluster analysis

Cluster analysis was performed for grouping differentially expressed genes exhibiting similar expression patterns. Differentially expressed genes were analyzed using the HPCluster program [14]. HPCluster is a two-stage algorithm: the first stage is based on BIRCH (Balanced Iterative Reducing and Clustering using Hierarchies), whereas the second stage is a conventional k-Means. With BIRCH, a tree of clustered features defining the partitioning of high-dimensional space was generated, followed by a conventional k-Means clustering of each cluster feature obtained with BIRCH.

### Gene ontology analysis

Associations of the differentially expressed genes with biological processes, molecular functions, and pathways were annotated using the PANTHER (Protein ANalysis THrough Evolutionary Relationships) classification system [15,16]. To determine whether the observed number of gene counts exceeded the expected counts, one-tailed *P *values for enrichment of a particular biological process, molecular function, or pathway were calculated using the standard Fisher exact test.

## Results

The present study was designed to define the changing gene expression profiles within the salivary glands of C57BL/6.NOD-*Aec1Aec2 *mice at five time points representing a pre-disease stage (4 weeks), the early pre-clinical stage (8 weeks), the initial influx of leukocytes into the salivary glands (12 weeks), the early clinical phase of autoimmunity (16 weeks), and the early onset of clinical SjS-like disease characterized by secretory dysfunction (20 weeks). The C57BL/6.NOD-*Aec1Aec2 *mouse is a model of primary SjS in which the *Idd3 *region of chromosome 3 and the *Idd5 *region of chromosome 1 derived from the NOD mouse were bred into the non-autoimmune C57BL/6 mouse, resulting in an SjS-like disease susceptibility that mimics both the pathophysiological characteristics and reduced secretory responses observed with NOD mice during development and onset of disease [4,17,18]. In C57BL/6.NOD-*Aec1Aec2*, *Aec1 *corresponds to *Idd3 *and *Aec2 *corresponds to *Idd5 *[18].

For the present study, we elected to begin the analyses at 4 weeks of age despite the fact that some intrinsic glandular changes occur in the salivary glands of NOD mice at an earlier age, especially around the time of birth [7]. However, salivary glands in C57BL/6.NOD-*Aec1Aec2 *mice appear normal by histology and protein secretion profiles at 4 weeks of age; as a result, the 4-week-old time point was established as the baseline for temporal analyses in these studies. Furthermore, we hypothesized that, by examining five time points spaced 4 weeks apart, genes identified as being differentially expressed after 4 weeks would correlate with one or more manifestations of aberrant glandular homeostasis, initiation of autoimmunity, and subsequent onset of salivary gland secretory dysfunction. In addition, by carrying out parallel analyses using salivary glands from the parental C57BL/6J strain, we should be able to identify genes that might be differentially expressed due merely to the natural aging process, thereby eliminating these from further consideration as disease-associated genes.

### Differential gene expressions in salivary glands of C57BL/6.NOD-*Aec1Aec2 *mice during development and onset of Sjögren's syndrome-like disease

**Figure 1:**
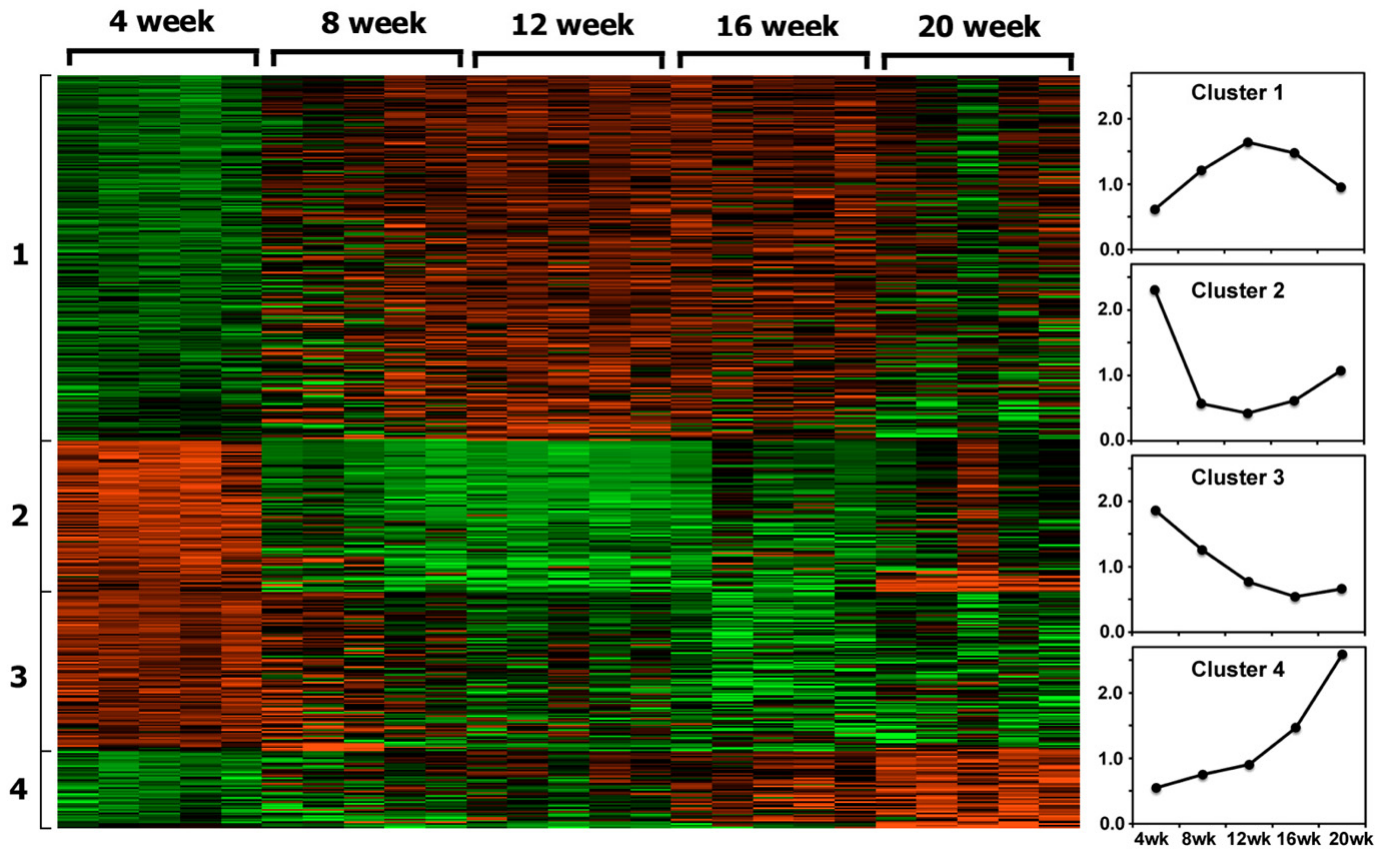
Expression profiles of differentially expressed genes depicted by Heatmap and HPCluster analyses. Heatmap of differentially expressed genes (n = 480) in the salivary glands of 25 individual C57BL/6.NOD-*Aec1Aec2 *male mice (n = 5 mice per age group) at 4, 8, 12, 16, and 20 weeks of age, grouped into four clusters based on temporal expression profiles (left panel). Upregulated gene expressions are shown in red, and downregulated gene expressions are shown in green. Based on HPCluster analyses, the averaged gene expression patterns for each of the four clusters are graphically modeled as temporal plots over the five time points measured (right panels). Aec, autoimmunexocrinopathy; NOD, non-obese diabetic.

With a statistical discrimination *P *value set at less than 0.05, LIMMA software and B-statistics analyses identified 480 specific genes as being differentially expressed in the salivary glands of C57BL/6.NOD-*Aec1Aec2 *mice during SjS disease development, despite the fact that many additional genes appeared to be differentially expressed at any particular time point. As illustrated in the heatmap shown in Figure 1 (left panel), these 480 genes can be compartmentalized into one of four highly reproducible clusters, each of which exhibits a specific temporal gene expression profile. In addition, each cluster can be graphically modeled as temporal plots (Figure 1, right panel), based on HPCluster analyses, showing the averaged gene expression patterns over the five time points. For quick verification of results obtained from the microarrays, four genes (*Ctsb*, *ApoE*, *Akt1*, and *Fdft1*) were selected randomly for semi-quantitative reverse transcriptase-PCR analysis as they represented genes that were expressed at high, intermediate, low, and depressed levels, respectively, in the salivary glands of C57BL/6J.NOD-*Aec1Aec2 *mice at various ages tested. The expression of these genes in the salivary glands relative to *G3pdh *(Additional data file 1) proved to be highly consistent with the relative expressions obtained from the microarrays, thus validating the relative expressions obtained with the current microarrays.

### Pathways, biological processes, and molecular functions of genes differentially expressed in salivary gland tissues

**Figure 2:**
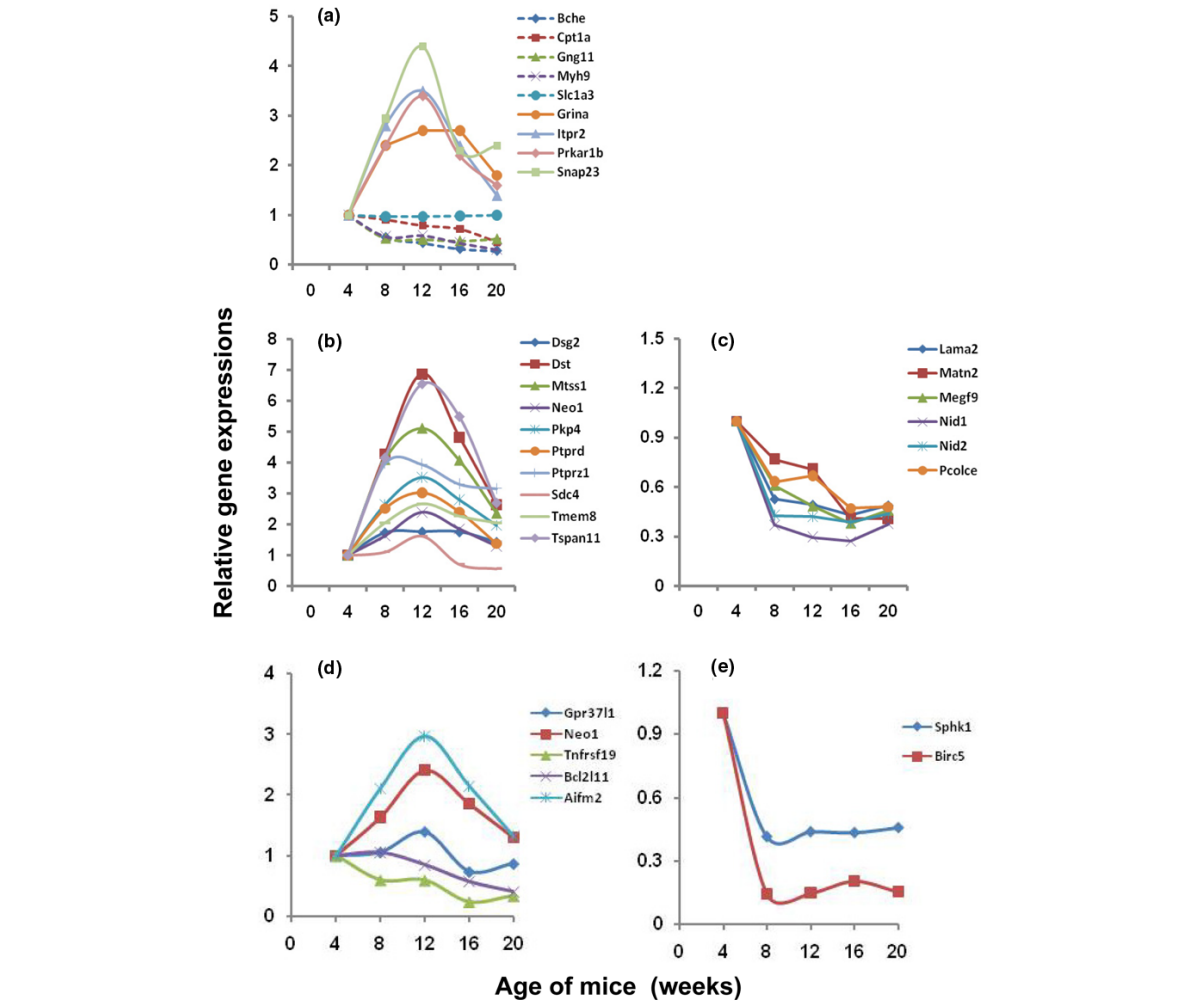
Representative temporal expressions of genes within biological pathways, processes, or molecular function, as identified by PANTHER (Protein ANalysis THrough Evolutionary Relationships). These include the muscarinic acetylcholine receptor signaling pathways **(a)**, collagen and collagen-associated **(b) **or non-collagen **(c) **genes associated with general cell adhesion, and genes associated with induction **(d) **and inhibition **(e) **of apoptosis.

By means of the PANTHER classification system, these 480 differentially expressed genes were categorized as being associated with specific biological pathways (Table 1), biological processes (Table 2), and/or molecular functions (Table 3). Several of the biological pathways identified might be anticipated as being directly involved with age-dependent aspects of normal development and activities; thus, it was not surprising that integrin signaling, vitamin B_6 _metabolism, p53-mediated transcription, fibroblast growth factor signaling, and hedgehog signaling pathways were also identified as being differentially expressed in parental, SjS-non-susceptible C57BL/6 mice (data not shown), suggesting that these pathways contain genes differentially expressed as a result of age and probably not disease. However, the specific pathway genes identified as differentially expressed in C57BL/6J parental mice were only occasionally the same genes as those differentially expressed in C57BL/6.NOD-*Aec1Aec2 *mice, despite being assigned to the same pathway(s). An example of this is demonstrated by the fact that, of the 21 integrin signaling pathway-associated genes, 13 encoded different collagen proteins in the salivary glands of C57BL/6.NOD-*Aec1Aec2 *mice, but only 4 of these 13 collagen genes were identified as differentially expressed in salivary glands of C57BL/6J mice (C.Q. Nguyen, A. Sharma, B.H. Lee, J.-X. She, R.A. McIndoe, A.B. Peck, unpublished data). Two pathways identified in the salivary glands as containing differentially expressed genes unique to the C57BL/6.NOD-*Aec1Aec2 *mice are the muscarinic acetylcholine receptor (mAChR) pathways. Loss of saliva secretion is thought to result, in part, from auto-antibodies reactive with the mAChRs [19-21]. In this association, four genes (*Snap23*, *Itpr2*, *Prkar1b*, and *Grina*) were upregulated with maximum expression levels occurring around 12 weeks of age; one gene (*Slc1a3*) remained unchanged, whereas four genes (*Bche*, *Cpt1a*, *Gng11*, and *Myh9*) showed progressive downregulation (Figure 2a). The latter four genes showed reduced levels of expression at 20 weeks of age, or the time that loss of saliva secretion is detected.

**Table 1:** Pathways represented in 480 differentially expressed genes with highest statistical discrimination

Classifications	Number of genes	Percentage of genes	*P *value
Pathways			
Integrin signaling pathway (P00034)	21	4.60%	9.92 × 10^-10^
Vitamin B6 metabolism (P02787)	3	0.70%	3.53 × 10^-5^
p53 pathway (P00059)	10	2.20%	4.77 × 10^-5^
Cell cycle (P00013)	4	0.90%	0.000843
Muscarinic acetylcholine receptor 2 and 4 signaling pathway (P00043)	5	1.10%	0.002843
Muscarinic acetylcholine receptor 1 and 3 signaling pathway (P00042)	4	0.90%	0.018809
Angiogenesis (P00005)	9	2.00%	0.019019
Metabotropic glutamate receptor group I pathway (P00041)	3	0.70%	0.020464
FGF signaling pathway (P00021)	6	1.30%	0.023515
Hedgehog signaling pathway (P00025)	3	0.70%	0.028308
VEGF signaling pathway (P00056)	4	0.90%	0.033627
PI3 kinase pathway (P00048)	5	1.10%	0.039324

FGF, fibroblast growth factor; VEGF, vascular endothelial growth factor.

**Table 2:** Biological processes represented in 480 differentially expressed genes with highest statistical discrimination

Classifications	Number of genes	Percentage of genes	*P *value
Biological process			
Cell structure and motility (BP00285)	40	8.70%	1.00 × 10^-6^
Other metabolism (BP00289)	27	5.90%	1.90 × 10^-6^
Cell cycle (BP00203)	35	7.60%	7.17 × 10^-6^
Cell adhesion (BP00124)	25	5.40%	5.23 × 10^-5^
Transport (BP00141)	39	8.50%	0.00059
Amino acid metabolism (BP00013)	11	2.40%	0.001705
Carbohydrate metabolism (BP00001)	19	4.10%	0.003036
Phosphate metabolism (BP00095)	6	1.30%	0.009946
Protein targeting and localization (BP00137)	8	1.70%	0.019608
Homeostasis (BP00267)	8	1.70%	0.02484
Oncogenesis (BP00281)	13	2.80%	0.030304
Apoptosis (BP00179)	14	3.00%	0.043403
Blood circulation and gas exchange (BP00209)	4	0.90%	0.048594

**Table 3:** Molecular functions represented in 480 differentially expressed genes with highest statistical discrimination

Classifications	Number of genes	Percentage of genes	*P *value
Molecular function			
Extracellular matrix (MF00178)	25	5.40%	1.24 × 10^-9^
Select regulatory molecule (MF00093)	38	8.30%	8.61 × 10^-5^
Isomerase (MF00166)	11	2.40%	9.04 × 10^-5^
Transporter (MF00082)	25	5.40%	0.000151
Oxidoreductase (MF00123)	23	5.00%	0.001595
Cytoskeletal protein (MF00091)	25	5.40%	0.002244
Kinase (MF00107)	22	4.80%	0.003857
Synthase and synthetase (MF00118)	9	2.00%	0.018598
Transferase (MF00131)	23	5.00%	0.041613
Protease (MF00153)	16	3.50%	0.047429

In addition to biological pathways, several biological processes (for example, cellular metabolism, blood circulation, and apoptosis) (Table 2) and molecular functions (for example, enzymatic activities) (Table 3) are identified via clustering of the differentially expressed genes. One important example involves the biological process of cell adhesion. Cell adhesion molecules are critical for gland development and subsequent remodeling of extracellular matrix during normal cellular homeostasis but may emerge as the consequence of pathogenic leukocytes being recruited into the salivary glands, resulting in glandular injury. A major feature of SjS is the presence of leukocyte infiltrates within the exocrine glands during the development and onset of disease, an event most likely mediated directly by the activation of adhesion molecules. As shown in Figure 2b, temporal analyses of genes encoding the adhesion molecules revealed that 10 of 16 genes encoding a variety of adhesion molecules (that is, *Dst*, *Tmem8*, *Pkp4*, *Mtss1*, *Dsg2*, *Ptprd*, *Neo1*, *Tspan11*, *Ptprz1*, and *Sdc4*) were upregulated, generally showing their highest expression levels around 12 weeks of age. In contrast, 6 of the 16 genes (that is, *Lama2*, *Matn2*, *Megf9*, *Nid1*, *Nid2*, and *Pcolce*) showed a progressive decrease in expression (Figure 2c). Although leukocyte infiltration of the salivary gland is first observed between 8 and 12 weeks of age, it is unknown which specific adhesion molecules are involved in these events.

A second set of genes considered important to the development of SjS-like disease and identified by their biological process involves apoptosis of acinar tissue. These genes can be separated into those that induce apoptosis (for example, *Gpr37l1*, *Neo1*, *Tnfrsf19*, *Bcl2l11*, and *Aifm2*) (Figure 2d) and those that inhibit apoptosis (for example, *Sphk1 *and *Birc5*) (Figure 2e). Interestingly, two genes, *Aifm2 *and *Neo1*, were upregulated showing maximum expression levels at 12 weeks of age, the *Gpr37l1 *gene showed little change over time, and the remaining genes, including *Sphk1 *and *Birc5 *encoding for inhibitory factors, were each downregulated showing a consistent temporal decrease in expression levels.

### Clustering of biological processes with identification of immuno-pathophysiological processes underlying Sjögren's syndrome-like disease

**Figure 3:**
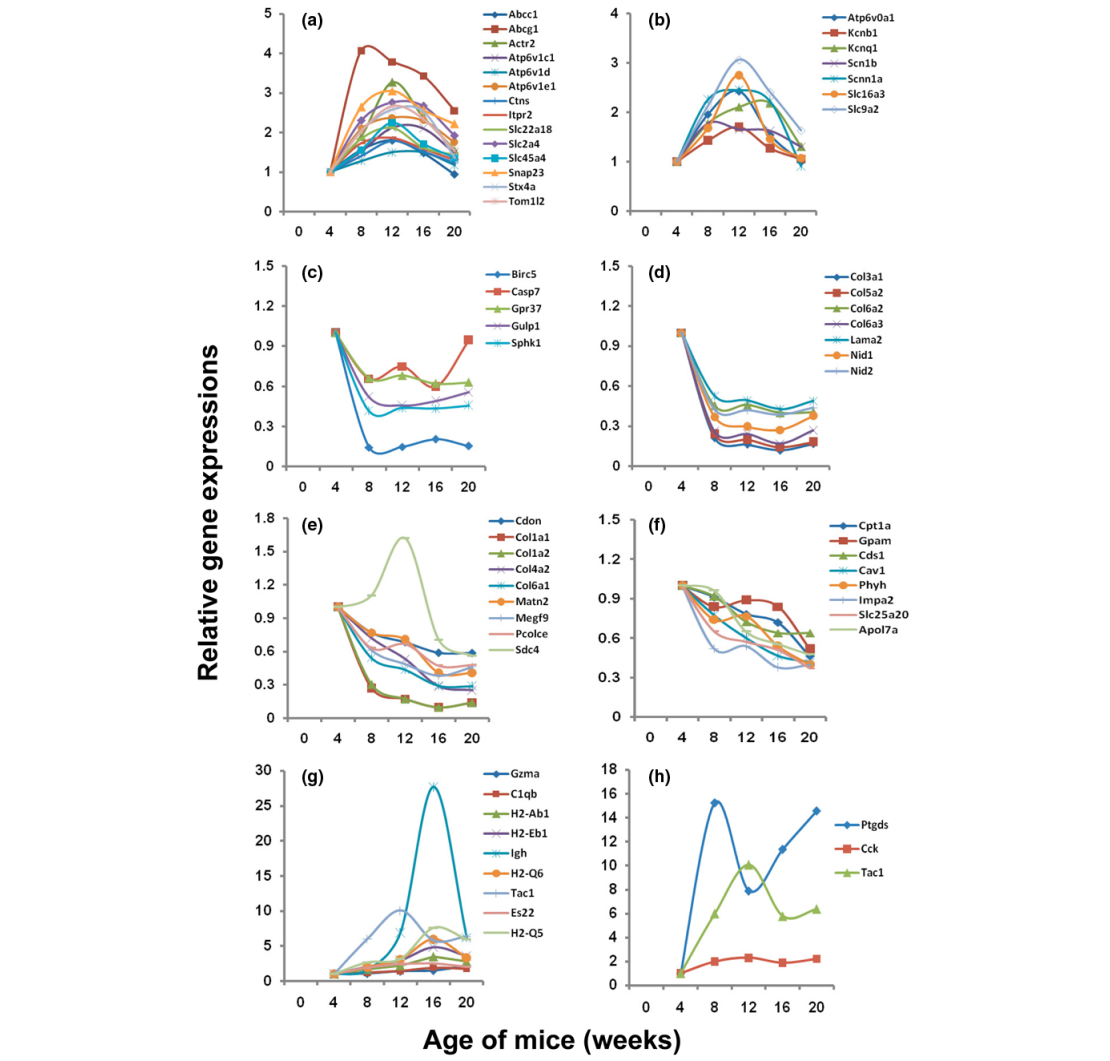
Examples of temporal changes in expression levels of selected genes associated with each of the four sets of genes identified by cluster analysis. Genes include those present in metabolite transport **(a) **and voltage-gate channels **(b) **of cluster 1, apoptosis **(c) **and adhesion **(d) **of cluster 2, adhesion **(e) **and fatty acid and lipid metabolism **(f) **of cluster 3, immunity **(g)**, and muscle cell contraction **(h)**. Each gene profile is presented as a pair-wise comparison of expressions in the salivary glands of C57BL/6.NOD-*Aec1Aec2 *mice at 8, 12, 16, and 20 weeks of age relative to 4 weeks of age. Aec, autoimmunexocrinopathy; NOD, non-obese diabetic.

As illustrated by the heatmap in Figure 1, four distinct clusters of genes showing comparable temporal gene expression profiles were established by HPCluster software analyses. To identify the various biological processes linked to genes grouped within the individual clusters, gene ontology analyses were performed separately for each cluster, and the results are presented in Table 4. Again, biological and molecular processes were considered statistically significant if they reached a *P *value of less than 0.05. Cluster 1, consisting of 233 genes, exhibits a temporal profile characterized by upregulated gene expressions in salivary glands of C57BL/6.NOD-*Aec1Aec2 *mice between 8 and 16 weeks of age, with the vast majority of genes showing maximal expressions at 12 weeks of age. Genes of cluster 1 belong to pathways whose functions are linked to normal metabolic functions, metabolite or ion transport and trafficking, and glandular integrity. The gradual loss of these activities after the 16-week time point most likely reflects the gradual onset of glandular pathology and dysfunction. This period in development of SjS-like disease represents the early phase of immunological activity in the salivary gland, yet preceding the onset of clinical disease; thus, it is not surprising that the differentially expressed genes are involved in either metabolic or secretory functions, most likely demonstrating attempts to balance injury, repair, and compensatory cellular activities. Genes associated generally with the transport of metabolites (for example, *Abcg1*, *Ctns*, and *Slc2a4*) or anions and cations (for example, *Abcc1*, *Atp6v*, and *Slc22a18*) and with voltage-gated channels (for example, *Kcnb1 *and *Scn1b*) illustrate this point, as presented in Figures 3a and 3b, respectively. One gene of particular interest is *Kcng1*, whose gene product binds with the gene product of *Kcne1 *to form a voltage-gated potassium channel regulator that may be functional in muscarinic receptors [22].

**Table 4:** Biological processes of differentially expressed genes of each cluster

Classification of clustered genes	Number of genes	Percentage of genes	*P *value
Cluster 1 (233 differentially expressed genes)			
Other metabolism (BP00289)	15	6.70%	8.09 × 10^-5^
Carbohydrate metabolism (BP00001)	13	5.80%	0.000695
Cell structure and motility (BP00285)	19	8.50%	0.000846
Transport (BP00141)	21	9.40%	0.003032
Phosphate metabolism (BP00095)	4	1.80%	0.011909
Cell cycle (BP00203)	14	6.30%	0.019047
Protein targeting and localization (BP00137)	5	2.20%	0.023674
Intracellular protein traffic (BP00125)	14	6.30%	0.025339
Cluster 2 (96 differentially expressed genes)			
Cell cycle (BP00203)	14	6.30%	3.05 × 10^-6^
Cell proliferation and differentiation (BP00224)	9	2.70%	0.004340
Cell adhesion (BP00124)	7	4.00%	0.005309
Cell structure and motility (BP00285)	9	8.50%	0.009058
Oncogenesis (BP00281)	5	0.90%	0.016163
Apoptosis (BP00179)	5	2.20%	0.028634
Homeostasis (BP00267)	3	1.80%	0.034910
Cluster 3 (102 differentially expressed genes)			
Amino acid metabolism (BP00013)	6	6.10%	0.0002
Cell adhesion (BP00124)	9	9.20%	0.0004
Cell structure and motility (BP00285)	11	11.20%	0.0012
Lipid, fatty acid, and steroid metabolism (BP00019)	9	9.20%	0.0024
Other metabolism (BP00289)	6	6.10%	0.0172
Oncogenesis (BP00281)	5	5.10%	0.0190
Developmental processes (BP00193)	13	13.30%	0.0475
Cluster 4 (49 differentially expressed genes)			
Immunity and defense (BP00148)	9	20.00%	0.0020
Muscle contraction (BP00173)	3	6.70%	0.0030

Cluster 2, comprised of 96 genes, exhibits expression profiles characterized by genes whose expressions are highly upregulated in the salivary glands of C57BL/6.NOD-*Aec1Aec2 *mice at 4 weeks of age but downregulated thereafter. Genes of this cluster appear to be involved primarily with maintenance of glandular structure, especially cellular processes such as cell cycling and cell proliferation/differentiation. However, inclusion of genes associated with apoptosis and cell adhesion may point to early events that indicate pathophysiological activities such as changes in glandular homeostasis, increased cell death, and impaired structural integrity. As presented in Figure 3c, five genes involved in apoptosis were identified as differentially expressed. Of note, at 20 weeks of age, the apoptosis-inducing factor caspase-7 shows an upregulated expression in the salivary glands, consistent with the concept that this is a second wave of apoptosis occurring at the time of immune attack possibly initiated by early apoptotic events seen at 4 weeks of age. At the same time, BIRC5, an anti-apoptotic factor, is strongly downregulated after the 4-week time point. Another set of cluster 2-associated genes that encodes cell adhesion molecules includes several collagen genes plus the laminin B gene (*Lama2*) and two nidogen genes (*Nid1 *and *Nid2*) (Figure 3d). Nidogen is thought to connect the laminin and collagen networks to stabilize basement membranes [23].

Cluster 3, consisting of 102 genes, contains genes involved in normal cellular physiology, but also cell adhesion, lipid/fatty acid/steroid metabolism, and oncogenesis, three processes that have been linked to autoimmunity in SjS. Genes in cluster 3 exhibit expression profiles similar to that of cluster 2, but distinguished in part by the fact that the decline in gene expression is less pronounced and generally remains downregulated through the onset of glandular dysfunction (that is, 20 weeks of age). Like cluster 2 genes, those of cluster 3 encode for a set of adhesion molecules (Figure 3e). This set of genes consists of a distinct set of collagen genes, the matrilin gene (*Matn2*), the multiple EGF-like domain 9 gene (*Megf9*), the procollagen-C endopeptidase enhancer gene (*Pcolce*), and the Syndecan 4 gene (*Sdc4*). Matrilin is involved in extracellular matrix assembly, MEGF9 is a trans-membrane molecule involved in neural development, and PCOLCE enhances procollagen-C endopeptidase cleavage of procollagen to form fibrillar collagen type 1. Expressions of these genes tend to mimic those seen for the cluster 2-associated adhesion molecules with the exception of *Sdc4*, which is upregulated through 12 weeks of age before being downregulated. *Sdc4 *encodes for an adhesion proteoglycan expressed on epithelial cells involved in growth factor receptor signaling. A second set of cluster 3-associated genes of particular interest involves potential impairment of lipid, fatty acid, and steroid hormone metabolism (Figure 3f). Of special interest is *Cav1*, which encodes for caveolin-1, a molecule associated with the integrity of lipid rafts, but also the Erk signaling pathway via Ras/Raf-1. Although recent studies have suggested that impairment of lipid metabolism and transport is restricted mostly to ocular surface-related disease and not salivary gland disease, these new data point to the possibility that lipid and fatty acid metabolism plays an important role in salivary gland dysfunction and onset of xerostomia as well. This would be consistent with our recent work [24,25] in which gene mapping data indicate that the SjS-susceptibility region *Aec2 *in C57BL/6.NOD-*Aec1Aec2 *mice contains multiple genes that regulate homeostasis of fatty acids, high-density lipids, and lipoproteins.

In contrast to genes associated with clusters 1 through 3, those associated with cluster 4 represent a limited subset of 49 genes whose maximal expressions in the salivary glands occur between 16 and 20 weeks of age, the time at which the covert autoimmunity finally results in measurable dysfunction of salivary and lacrimal gland secretions in these mice. As might be expected, the genes in cluster 4 are linked predominantly to immunity (Figure 3g), with a lesser number linked to muscle contraction (Figure 3h). The latter set of genes correlates with altered neural stimulation and direct loss of secretory function. Examination of the cluster 4-associated genes indicates that several of the identified genes encode for major histocompatibility class I (*H2q5 *and *H2q6*) and class II (*H2ab *and *H2eb*) products, a complement component (*C1qb*), an immunoglobulin heavy chain (*Igh*), the apoptosis-inducing protease granzyme A (*Gzma*), and preprotachykinin (*Tac1*). Tachykinin is involved not only in inflammatory responses, but in neural stimulation as well, thereby bridging inflammation to muscle contraction [26].

### Phase-specific gene expressions in the salivary glands of C57BL/6.NOD-*Aec1Aec2 *mice during development of Sjögren's syndrome-like disease

**Figure 4:**
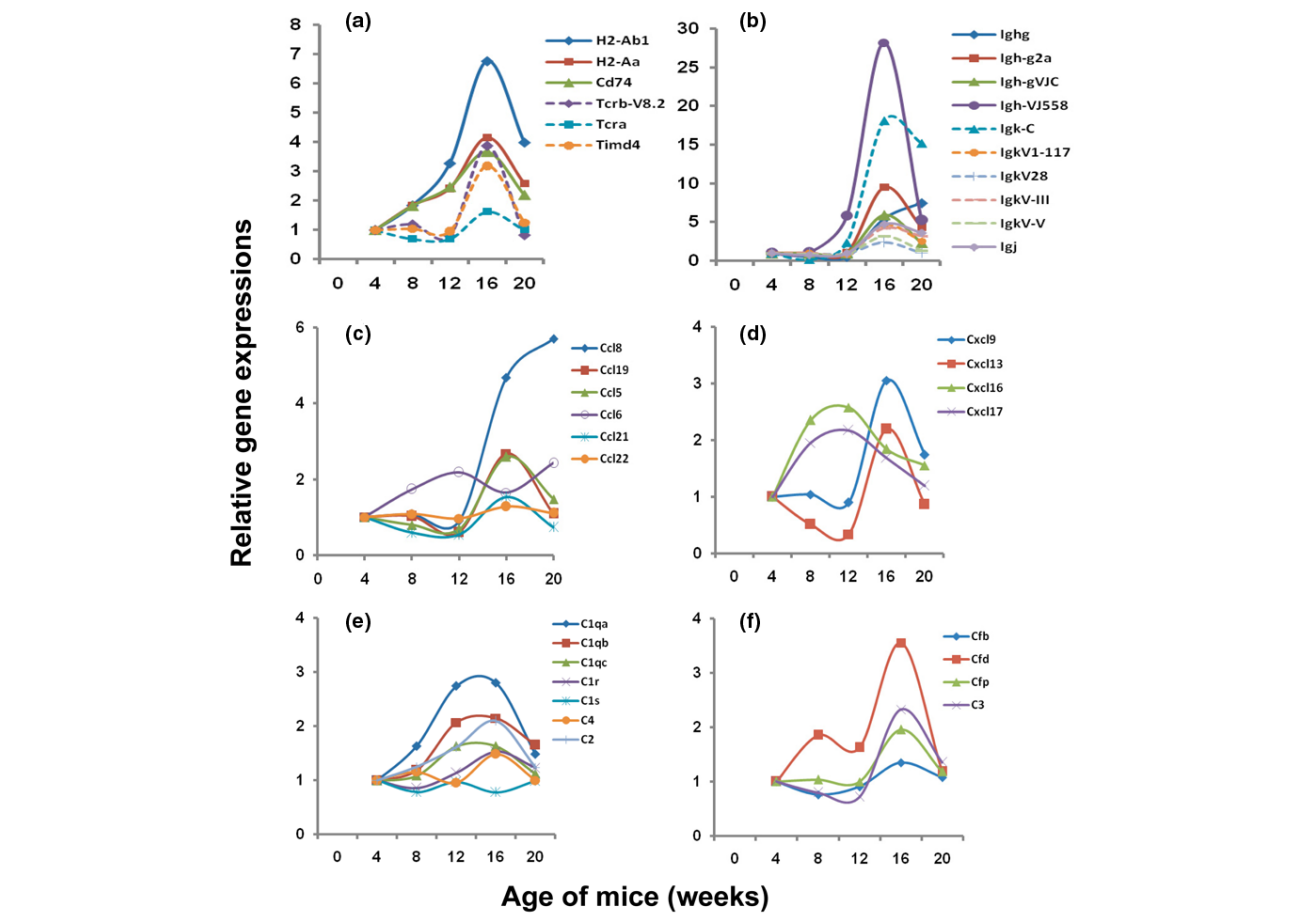
Temporal expressions of genes associated with autoimmunity in the salivary glands of C57BL/6.NOD-*Aec1Aec2 *mice at 8, 12, 16, and 20 weeks of age relative to 4 weeks of age, including antigen presentation **(a)**, immunoglobulin synthesis **(b)**, chemokine production **(c, d)**, C1q genes **(e)**, and the alternate complement pathway **(f)**, as identified by pair-wise analysis but not Linear Models for Microarray Analysis (LIMMA) and B-statistics. Aec, autoimmunexocrinopathy; NOD, non-obese diabetic.

As described above, both functional pathways and biological processes can be identified through the clustering of differentially expressed genes based on their temporal profiles over the five selected time points examined. Since these microarray data measure differential gene expressions covering the majority of the mouse genome and, at the same time, span temporally the progressive development and early onset of autoimmune-mediated xerostomia in salivary glands of C57BL/6.NOD-*Aec1Aec2 *mice, each represented gene can be examined individually for its expression profile, even when not identified as being statistically significant using LIMMA and B-statistics. When analysis is conducted in this manner, a marked increase in the number of individual genes that exhibit distinct expression kinetics occurs and is often associated with a particular phase of disease. This latter point is clearly demonstrated when one expands the gene set involved in immunity beyond the genes presented in Figure 3g. Using a pair-wise analysis, we have uncovered several genes that encode factors important in T cell-antigen-presenting cell interactions (Figure 4a), B-cell antibody production (Figure 4b), members of the chemokine-ligand families, CCL and CxCL (Figure 4c,d), and complement-associated factors (Figure 4e,f) that show marked differential expressions during development of SjS-like disease. These data indicate that the expression profiles for the chemokine, T cell-associated, and immunoglobulin genes precisely mimic the temporal appearance of macrophages/dendritic cells and of T and B lymphocytes into the salivary glands, as determined by immuno-histochemical staining. More specifically, genes associated with macrophages and dendritic cells exhibit upregulation as early as 8 weeks of age whereas T and B cell-associated genes exhibit upregulated expressions around 12 weeks of age.

**Figure 5:**
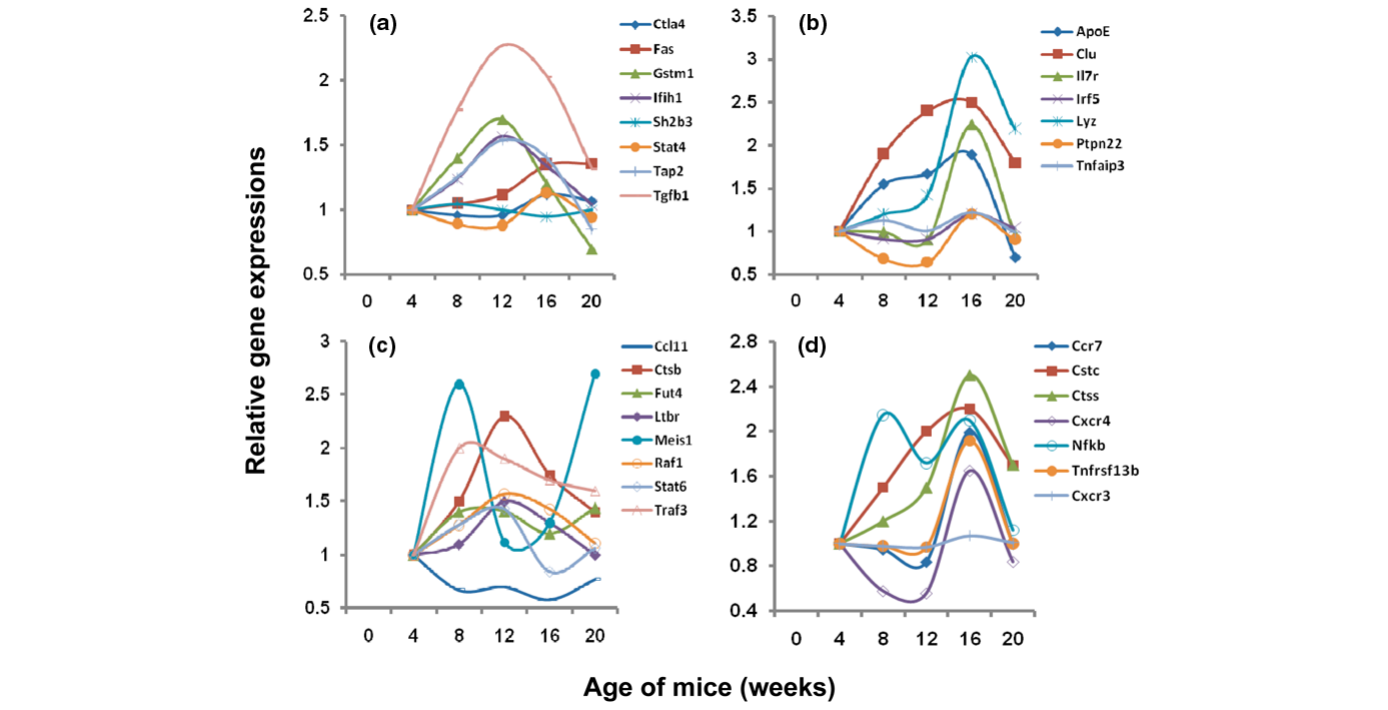
Differential expression of genes reported to be associated with connective tissue autoimmunity in humans and/or mouse models. Temporal changes in gene expressions in the salivary glands of C57BL/6.NOD-*Aec1Aec2 *mice for genes that are reportedly associated with Sjögren's syndrome, systemic lupus erythematosus, and/or rheumatoid arthritis in humans **(a, b) **and genes considered important for disease development in the salivary glands of non-obese diabetic (NOD) and NOD-derived mice **(c, d)**. Expression profiles are presented for these genes at 8, 12, 16, and 20 weeks of age relative to 4 weeks of age. Aec, autoimmunexocrinopathy.

Although studies linking specific genes with SjS remain limited, several genes and/or gene products have been reported as being associated with either SjS or diseases linked to SjS, such as systemic lupus erythematosus (SLE) and rheumatoid arthritis. These include such genes as *ApoE *[27], *Clu *[28], *Ctla4 *[29], *Fas/Fasl *[30], *Gstm1 *[31], *Il7r *[32], *Ifih1 *[33], *IgG *[34], *Irf5 *[35], *Lyzs *[34], *Mbl *[36], *Ptpn22 *[37], *Sh2b3 *[38], *Stat4 *[39], *Tap2 *[34], *Tgfβ1*, *Tnfa *[40], and *Tnfaip3 *[41]. At the same time, a growing list of genes and/or gene products has been reported as being associated with SjS-like disease in mouse models [10,42]. These genes include *Abpb*, *ApoA1*, *Baff (Blys)*, *Ccl11*, *Ccr7*, *Ctss*, *Ctsb*, *Cstc*, *Cxcr3*, *Cxcr4*, *Egf*, *Fgl*, *Fut4*, *Il10r*, *Isg*, *Ltb*, *Ltbr*, *Meis1*, *Nfkβia*, *Pgf*, *Rac1*, *Raf1*, *Socs3*, *Stat6*, *Traf3*, *Tnfrsf13*, and *Vcam1*. Despite the fact that C57BL/6.NOD-*Aec1Aec2 *mice represent a single individual genetically, a surprisingly high number of these genes, whether associated with human disease or disease in mouse models, exhibited specific temporal changes in their expression profiles when analyzed using a pair-wise comparison, as presented in Figure 5.

## Discussion

In the present study, we used microarray technology to identify genes whose temporal expressions are differentially regulated during development of sialadenitis and xerostomia in the C57BL/6.NOD-*Aec1Aec2 *mouse model of primary SjS. The use of C57BL/6.NOD-*Aec1Aec2 *mice offers two advantages for microarray studies: first, the C57BL/6J background of C57BL/6.NOD-*Aec1Aec2 *mice eliminates features of NOD mice which complicate interpretation of potential underlying genetic and pathophysiological causes of autoimmunity, and second, non-autoimmune C57BL/6J parental mice provide an excellent comparative control identifying normal physiological changes. Development of SjS-like disease in C57BL/6.NOD-*Aec1Aec2 *mice progresses through several sequential, yet continuous, covert pathological stages, resulting eventually in the onset of overt clinical manifestations. Our extensive studies with C57BL/6.NOD-*Aec1Aec2 *mice have defined biological and immunological features associated with various stages of SjS-like disease [4], thereby establishing a basis for correlating gene expressions and pathology.

Results presented herein, similar to microarray results recently reported for the lacrimal gland [9], indicate that HPCluster analysis of the microarray data identifies multiple sets of genes whose associated pathways correlate with concepts currently hypothesized to explain the early pathophysiological processes and subsequent autoimmunity. In this regard, our statistical analyses identified 480 genes that were differentially expressed in the salivary glands over the five ages examined, although few of these genes actually reside within either the *Aec1 *or *Aec2 *genetic regions. Since the vast majority of these 480 genes are scattered throughout the genome, identifying primary candidate genes regulating development of SjS-like disease versus secondary genes representing either activations of downstream events or functionally linked molecular interactions poses a daunting task. Nevertheless, the present results appear to provide validation for the genomic microarray approach in understanding the underlying immuno-pathophysiological features of SjS.

This study also provides a global genomic analysis of differentially expressed genes, permitting an expansive overview of biological processes and gene interactions revealing potentially important pathways even when many genes within a particular pathway may not exhibit altered expression or be involved in normal cellular functions. Examples of this include the upregulation of genes involved in apoptosis, cell adhesions, or homeostasis of lipid, lipoprotein, and fatty acid metabolism, three biological processes central to salivary and lacrimal gland functions. In addition, this study revealed individual genes that exhibit differential expressions during specific phases of disease, pointing to additional interactive pathways involved in pathological events. Examples of such genes would include those encoding for chemokines, complement factors, and T- and B-cell signaling.

Although microarray data permit the identification of genes that are differentially expressed, perhaps one of the more interesting results highlighted by the present study, and in support of our previous studies [9,10], is the ability of the microarray analyses to confirm a rapidly changing gene expression profile during the chronic progression of disease development and subsequent salivary gland dysfunction. While considerable emphasis is placed on genes that are upregulated as being involved in this pathogenesis, many genes exhibit statistically significant downregulated differential expressions, as depicted in clusters 2 and 3 of the heatmap and in the pair-wise analyses. We suspect that this indicates two important events. First, at 4 weeks of age, major changes in salivary gland homeostasis depicting normal, age-related cellular processes are occurring, thus resulting in decreased expressions of these genes at the later ages. Second, development of pathological conditions within the salivary gland results in loss of cellular functions, thereby decreasing gene expressions of many biological processes. Nevertheless, the largest cluster of genes (cluster 1) identified differentially expressed genes upregulated with maximal expression levels at 12 to 16 weeks of age, or the time of detectable inflammation. This would indicate that numerous aberrant biochemical and physiological activities are occurring prior to both autoimmunity and overt disease onset. Finally, except for genes associated mainly with inflammation and autoimmunity, most genes are no longer upregulated at the onset of clinical disease.

Data analyses in the present study used two distinct methods. The first was identification of genes differentially expressed based on statistical evaluations across development of disease. For the present study, we specifically chose a cutoff value of B-statistics *P *value of 0.05, which identified 480 genes with a probability of greater than 80% that each gene is differentially expressed. These were considered genes of interest identified in an unbiased manner. The second process, however, was to perform pair-wise analyses in which a gene's relative expressions at 8, 12, 16, and 20 weeks were compared with its expression at 4 weeks, an age point arbitrarily set as pre-disease. This analysis was used to identify genes whose differential expressions might correlate with a specific phase of SjS-like disease and indicate a specific altered biological process.

A concern, and possible weakness, in these microarray analyses is whether important data are missed when differential gene expressions are determined by statistical measurements or pair-wise analyses. An example of this would be the detected expressions of chemokines and their receptors, a large family of proteins that regulate leukocyte trafficking to tissue sites. It is assumed that in SjS small numbers of macrophages along with dendritic cells are the first leukocytes to enter the salivary glands, acting to recruit the T and B lymphocytes that subsequently form lymphocytic foci commonly seen in the exocrine glands of SjS patients and animal models. This raises the question of whether the low numbers of these cell populations express sufficient levels of mRNA transcripts for detection. In the present study, we were able to detect expression of several CXC and CC ligand genes in the salivary glands starting at 8 weeks of age, the precise time when leukocytes begin entering the exocrine tissues in large numbers. The relatively limited profile of detectable chemokines probably indicates a highly restricted transcript expression. Of interest, the first CXC ligand chemokine gene detected is *Cxcl16*, an interferon-gamma-regulated chemokine whose product attracts natural killer (NK) cells and memory CD4^+ ^memory T cells (possibly T_H_17 cells). This is subsequently followed by detectable *Cxcl9*, whose product attracts T_H_1 cells, and *Cxcl13*, whose product attracts B1 and B2 B lymphocytes.

Recently, Delaleu and colleagues [42] reported that several CCL chemokines, including CCL-2, CCL-5, CCL-7, CCL-9, CCL-19, and CCL-22, were differentially expressed in sera and/or saliva of NOD mice at onset of SjS-like disease when compared with levels observed in BALB/c mice, suggesting that these proteins are biomarkers since they correlated with the state of hyposalivation. In the present study, we found *Ccl5 *(coding for Rantes), *Ccl8 *(coding for MCP-2), *Ccl19 *(coding for MIP-3), and possibly *Ccl21 *and *Ccl22 *to be upregulated. The most prevalent *Ccl *gene transcript in the salivary glands was *Ccl8*, whose product is a chemoattractant for monocytes and possibly NK and T cells. *Ccl6*, which shows a slight bi-modal expression and is clearly expressed prior to the other *Ccl *genes, is involved in myeloid cell differentiation; thus, this chemokine appears to be expressed in concert with *Cxcl16*. Although our microarray data do not match perfectly with the results reported by Delaleu and colleagues [42], it must be remembered that the latter study was carried out with fluids obtained from NOD mice predisposed for three autoimmune diseases: SjS, T1D, and possibly thyroiditis.

**Figure 6:**
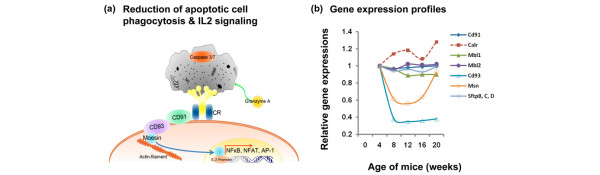
Temporal changes in gene expressions in the salivary glands of C57BL/6.NOD-*Aec1Aec2 *mice for genes associated with clearance of apoptotic cells. **(a)** Model of the proposed mechanism for the (lack of) clearance of apoptotic cell debris by phagocytic cells, resulting in reduced interleukin-2 (IL-2) production by phagocytic cells. **(b)** Expression of genes associated with apoptotic cell clearance. Expression profiles are presented for these genes at 8, 12, 16, and 20 weeks of age relative to 4 weeks of age. Aec, autoimmunexocrinopathy; AP-1, activation protein-1; CR, complement receptor; NFAT, nuclear factor of activated T cells; NF-κB, nuclear factor-kappa-B; NOD, non-obese diabetic.

Using pair-wise analyses, we have identified many genes that show temporal changes in expressions correlating with specific phases of SjS. This is clearly demonstrated by considering the profiles of complement component genes. Of particular interest is the complement factor C1q. Polymorphisms present in C1q have been shown to correlate with SLE in humans [43], but the role of complement in SjS remains highly speculative. Our recent studies in NOD and C57BL/6.NOD-*Aec1Aec2 *mice indicate a crucial role for C3 in the development of salivary and lacrimal gland dysfunction [44]. Results from the present microarray study indicate that transcripts of *C1qα*, *C1qβ*, and *C1qγ*, whose products form the core of C1q, are highly upregulated, showing maximal levels between 12 and 16 weeks of age. Whereas transcription of *C1r *was slightly elevated, transcription of *C1s *was slightly depressed. Since C1s is essential for activation of the classical complement pathway, we hypothesize that the complement membrane attack complex plays little or no role in SjS-associated salivary gland disease, a concept supported by the fact that expressions of *C6 *through *C9 *transcripts were unchanged, plus the high transcript levels of both *Clu *(clusterin) and *CD59 *(protectin), two complement membrane attack complex inhibitors. In contrast, genes comprising the alternate pathway (that is, *Cfb*, *Cfd*, *Cfp*, and *C3*) were all upregulated, suggesting that the alternate pathway may be active in the disease process. At the same time, C1q may be upregulated in response to a decreased efficacy in the clearance of apoptotic cells/debris that could affect tissue homeostasis, antigen presentation, and subsequent regulatory T (T_reg_) cell activation. In addition to C1q, factors involved in the clearance of apoptotic debris by phagocytes include CD91/LRP-1, CD93, and calreticulin, as modeled in Figure 6a. In brief, C1q is one of the known molecules responsible for binding to apoptotic cells in order for efficient clearance, especially by macrophages [45,46]. Whether this function requires the C1r and C1s subunits is unclear, but deficiencies in a subunit of C1q can increase susceptibility to autoimmunity [47]. The interaction between C1q and its receptors on phagocytes is mediated by calreticulin, a molecule that ultimately binds to the receptor, CD91/LRP-1, expressed in conjunction with CD93. CD93, in turn, is a cell surface molecule that is tethered to the cytoskeleton via moesin [45]. Moesin translocates to the nucleus during retinoic acid-induced differentiation, but this latter process involving RAR/PPAR-γ (retinoic acid receptor/peroxisome proliferator-activated receptor-gamma) signaling is hypothesized to be defective in this model [25]. Moesin, a member of the FERM (4.1 protein, ezrin, radixin, moesin) domain-containing family of proteins interacting with cytosolic tails of trans-membrane proteins, can activate the IL-2/IL-2R pathway [48], a system well known to be inefficient in maintaining T_reg _cells in this model of autoimmunity. Expression profiles for these genes, presented in Figure 6b, indicate that the transcript levels of these interactive factors are either downregulated or unchanged throughout the course of disease development, thereby suggesting that this mechanism of apoptotic cell clearance may not be functioning. Interestingly, other components known to participate in clearance of apoptotic cells (that is, the surfactant proteins SP-A, SP-B, SP-C, and SP-D or the mannose-binding lectin MBL) exhibit no temporal changes in gene expression levels.

Lastly, it is imperative to comment on the relevance of our microarray data with respect to human SjS and whether differentially expressed genes provide any clues to understanding the immuno-pathophysiological processes underlying SjS-like disease. As presented in Figure 5, our results demonstrated that a large number of genes/factors that have been reported to correlate with SjS or other rheumatic diseases in humans are also differentially expressed in the salivary glands of C57BL/6.NOD-*Aec1Aec2 *mice. Furthermore, the microarray study of Hjelmervik and colleagues [49], to date the most extensive microarray study of human SjS patients, shows a remarkable overlap with the current mouse studies, not necessarily between specific genes but within biological processes and functions of differentially expressed genes. These include upregulation of interferon-associated genes, antigen processing and presentation, T- and B-cell differentiation and functions, and apolipoproteins, plus downregulation of secretory and cell proliferation. Specific genes include *Il6*, *Cd74*, *CaII*, *Bcl2l2*, *Cxcl13*, and *Ccr7*. Thus, given the extent of the overlaps already seen, C57BL/6.NOD-*Aec1Aec2 *mice appear to offer a unique opportunity to identify genetic factors regulating processes leading to SjS.

In summary, our earlier microarray studies [9] with lacrimal glands from C57BL/6.NOD-*Aec1Aec2 *mice and now the present studies with salivary glands are permitting us to propose several new concepts pertaining to what molecular events may lead to SjS and SjS-like disease. These include (a) retarded maturation of myeloid cells, especially macrophages and dendritic cells, possibly due to depressed response mediated by low levels of retinoic acid receptors and PPAR-γ; (b) reduced efficacy of macrophages and dendritic cells in antigen presentation, possibly due to an overexpression of cathepsin-S, leading to increased auto-antigen presentation by B cells; (c) lack of regulation of the T_H_17 effector cell population by reduced activation of both T_reg_1 and T_Foxp3 _cells, possibly due to low levels of IL-2; (d) depositions of fatty acids and lipoproteins in the lacrimal glands, due to changes in lipid receptors and transporters; and (e) decreased efficacy in the clearance of apoptotic cells/debris, due to reduced signal transduction and activation of phagocytes via the C1q/CD91/CD93/moesin pathway. Functional studies should be able to determine which of these biological processes is (are) critical pathological entities for SjS.

## Conclusions

We have used a genomic approach to identify genes that are differentially expressed in the salivary glands during the development and early-onset phases of SjS-like disease of C57BL/6.NOD-*Aec1Aec2 *mice. This approach identified 480 genes that could be grouped into one of four expression patterns during development of disease. However, additional genes exhibited marked changes in their expressions during the time frame studied, based on simple pair-wise analyses. While a more complete analysis of these data will require considerable time, a number of expected and unexpected biological processes, signaling pathways, and potential dysfunctions have been identified. As might be predicted, virtually all biological processes during the early stages of disease (4 to 16 weeks of age) relate to altered cell functions, with inflammation- and autoimmunity-related processes appearing much later (16 to 20 weeks of age). Most importantly, these types of analyses permit construction of hypothetical models for SjS which now can be examined in greater detail *in vivo*, possibly confirming the identification of specific SjS-susceptibility candidate genes and their subsequent downstream molecular pathways.

## Abbreviations

Aec: autoimmunexocrinopathy; BIRCH: balanced iterative reducing and clustering using hierarchies; EGF: epidermal growth factor; Idd: insulin-dependent diabetes; IL: interleukin; LIMMA: Linear Models for Microarray Analysis; mAChR: muscarinic acetylcholine receptor; NK: natural killer; NOD: non-obese diabetic; PANTHER: protein analysis through evolutionary relationships; PCR: polymerase chain reaction; PPAR-γ: peroxisome proliferator-activated receptor-gamma; SjS: Sjögren's syndrome; SLE: systemic lupus erythematosus; T_H_: T helper (cell); T_reg_: regulatory T (cell).

## Competing interests

The authors declare that they have no competing interests.

## Authors' contributions

ABP designed the study and participated in the pair-wise analyses and in the preparation of the manuscript. CQN prepared the mRNA for microarray analysis and participated in the pair-wise analyses and in the preparation of the manuscript. BHL carried out the reverse transcriptase-polymerase chain reaction validation studies and participated in the preparation of the manuscript. JXS carried out the microarrays. AS and RAM provided the initial analyses of microarray data. All authors read and approved the final manuscript.

## Supplementary Material

Additional file 1 Verification of microarray data by RT-PCR. Four genes, *Ctsb, Apoe, Akt1 *and *Fdft1*, identified as displaying different levels of transcripts in the microarrays of salivary glands over time were selected for RT-PCR analyses. **A**. PCR band intensities visualized with ethidium bromide staining. **B**. Plot of PCR band intensities. **C**. Corresponding plot of transcripts as determined by microarray. *G3pdh *was used to control for fidelity of the RT-PCRs. As the band intensities of *G3pdh *remained constant over all the time points, the relative band intensities are not shown here. Click here for file
