# Supplementary material for: Differential gene expression in the salivary gland during development and onset of xerostomia in Sjögren's syndrome-like disease of the C57BL/6.NOD-Aec1Aec2 mouse
Source: Arthritis Res Ther. 2009 Apr 20;11(2):R56. doi: 10.1186/ar2676 (PMC2688207; doi:10.1186/ar2676)
Supplement: Additional file 1 — Verification of microarray data by RT-PCR. Four genes, Ctsb, Apoe, Akt1 and Fdft1, identified as displaying different levels of transcripts in the microarrays of salivary glands over time were selected for RT-PCR analyses. A. PCR band intensities visualized with ethidium bromide staining. B. Plot of PCR band intensities. C. Corresponding plot of transcripts as determined by microarray. G3pdh was used to control for fidelity of the RT-PCRs. As the band intensities of G3pdh remained constant over all the time points, the relative band intensities are not shown here. [file ar2676-S1.ppt]

## Slide 1
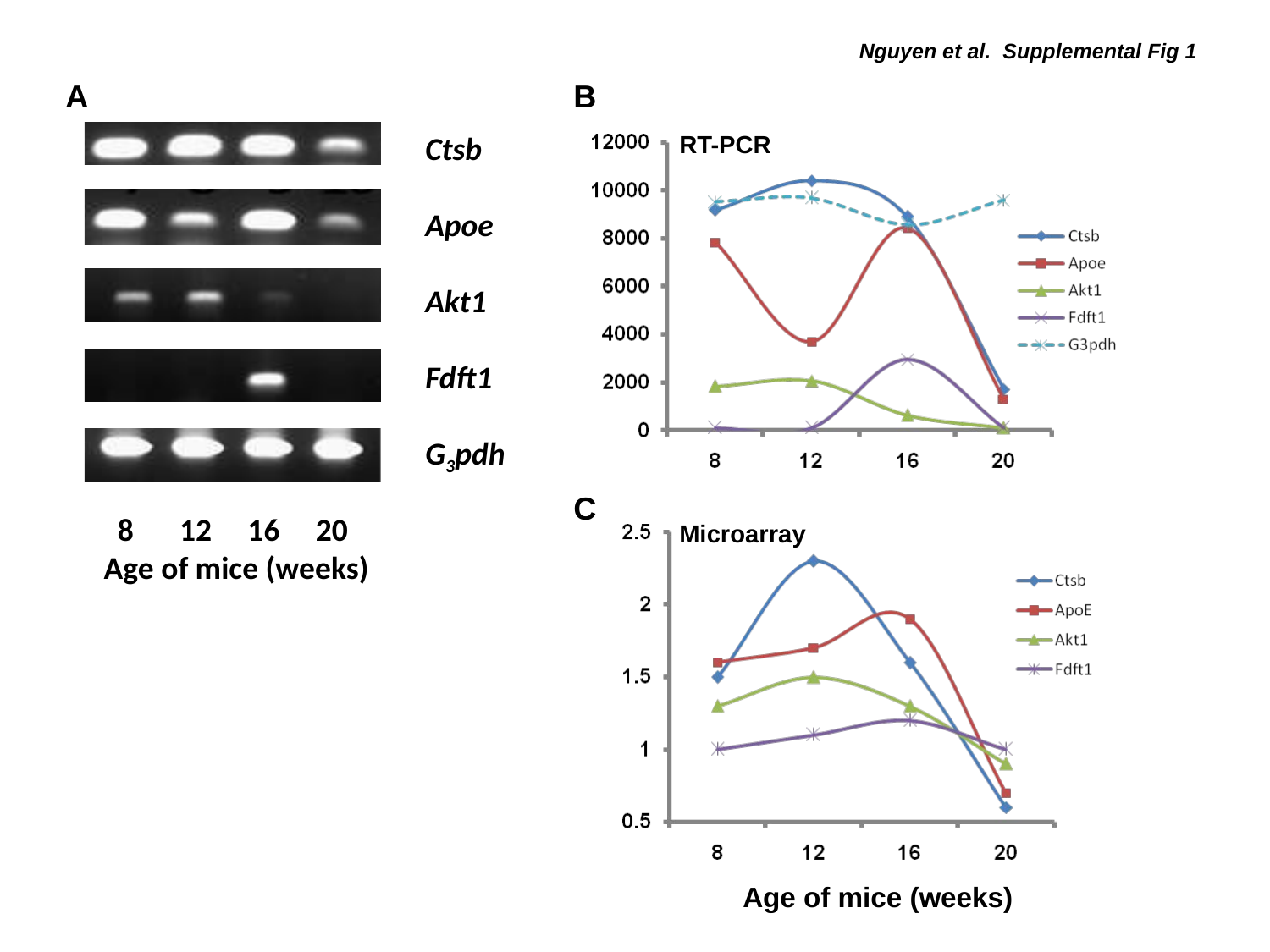

Nguyen et al. Supplemental Fig 1
A
B
Ctsb
Apoe
Akt1
Fdft1
G3pdh
 12 16 20
 Age of mice (weeks)
RT-PCR
C
Microarray
Age of mice (weeks)
